# Cervical Spine Injury Following Thoracic Spinal Fusion for Adolescent Idiopathic Scoliosis

**DOI:** 10.7759/cureus.5840

**Published:** 2019-10-05

**Authors:** Rahul G Samtani, James T Bernatz, Matthew A Halanski, Kenneth J Noonan

**Affiliations:** 1 Orthopaedics, University of Wisconsin Hospital and Clinics, Madison, USA; 2 Orthopaedics, University of Wisconsin, Madison School of Medicine and Public Health, Madison, USA

**Keywords:** adolescent, spinal cord injury, scoliosis, complication, cervical, paralysis, posterior spinal fusion, pediatrics, deformity

## Abstract

Spinal fusion for adolescent idiopathic scoliosis (AIS) can have many potential complications, including spinal cord injury. Most often, spinal cord injury occurs in the region of surgery due to direct mechanical trauma. Vascular compromise in this area may also occur due to a high degree of correction or excessive distraction of the spine. In these cases, the impairment of spinal cord function is often detected intraoperatively with spinal cord monitoring and confirmed in the immediate postoperative period. Injury to the spinal cord above the level of instrumentation is rare.

We review the clinical history and outcome of a female adolescent who underwent posterior spinal fusion (PSF) for AIS and developed a cervical spine injury 12 hours postoperatively. The patient is a 13-year old female who underwent PSF for AIS from T1 to L1 for progressive scoliosis measuring over 53 degrees in her right thoracic curve. During surgery, she had modest correction with minimal blood loss and with normal intraoperative motor evoked and somatosensory evoked potentials. The immediate postoperative examination was neurologically intact. Twelve hours later, she developed weakness and tingling in her right upper extremity. Magnetic resonance imaging (MRI) of the cervical spine demonstrated myelomalacia on the right side of the spinal cord at the C5-7 levels.

Cervical spine injuries are rare following lower-level fusions, however, these injuries can occur and it is important to be vigilant in monitoring patients for these symptoms. The exact mechanism is unknown and may include a combination of postoperative hypotension with altered vascular anatomy from cord stretch and abnormal cervical positioning.

## Introduction

Segmental instrumentation and spinal fusion can be recommended for growing patients with adolescent idiopathic scoliosis with progressive curves greater than 45-50 degrees. There are a multitude of risks associated with this treatment. Although uncommon, there is a risk for catastrophic neurological injury with subsequent deficits. A recent meta-analysis has shown the risk of neurologic injury to be in the range of 0.13%-0.93% [1-3]. Attempts to mitigate this risk are commonplace and include techniques such as intraoperative neuromonitoring with motor evoked potentials and somatosensory evoked potentials (MEPs and SSEPs). Neuromonitoring can immediately identify an intraoperative loss of function during the surgery and potentially allow for intraoperative adjustments, such as implant removal or curve correction modification that can avoid permanent disability [4].

Unfortunately, neuromonitoring techniques are rarely extended beyond the operating room and thus it is difficult to detect neurological complications that present in a delayed fashion. In a recent meta-analysis, delayed postoperative neurological deficits (DPND) were identified in .01% of cases [5]. The most common cause of the deficit was possible ischemic injury at the level of the fusion[5]. In our review of the literature, we found two other papers discussing cervical spinal cord injury ischemia secondary to thoracolumbar fusion [6-7]. We present a similar case to increase the body of literature available about this notable complication in a high-risk population.

## Case presentation

**Figure 1:**
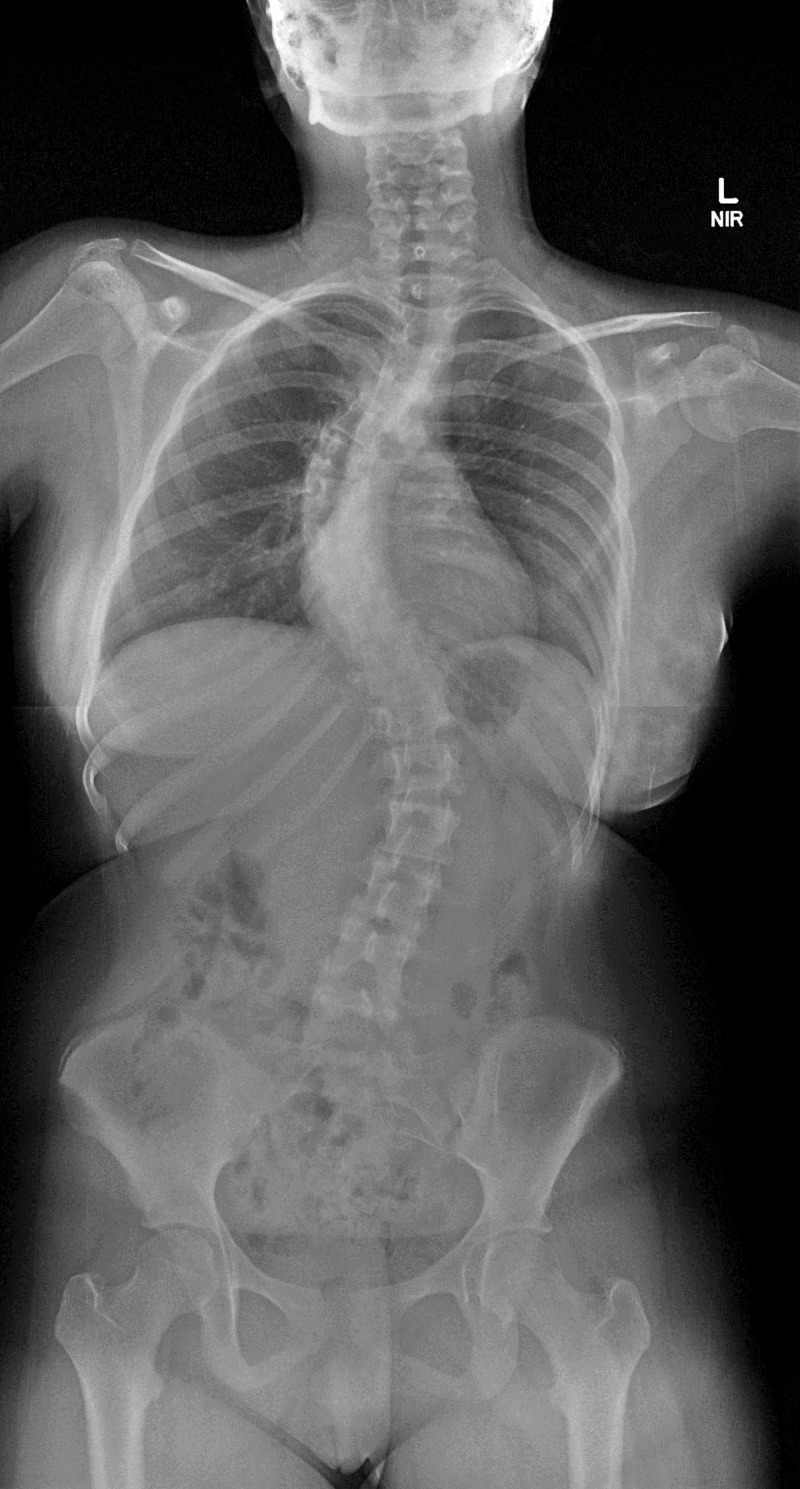
Preoperative AP radiograph demonstrating the degree of scoliosis curvature 53 degree right convex thoracic curve from T4-T10 and 49 degree left convex thoracolumbar curve from T11-L3 AP: anteroposterior

Our patient is a 13-year-old Risser 2 female with AIS consisting of a 53-degree right convex curvature curve from T4-T10 with a left convex thoracolumbar curve measuring 49 degrees from T11-L3 (Figure 1).

The patient reported occasional back pain throughout her life, but this was never activity limiting. She denied any neurological symptoms, including numbness or tingling in her extremities. Menarche was at age 11. An MRI was obtained of the entire cord to rule out an intrathecal process, such as Chiari malformation, syrinx, or cord tethering, and was found to be normal without signal abnormality within the spinal cord at any level.

After appropriate preoperative counseling, the patient and her family elected to undergo a posterior spinal fusion with instrumentation from T1-L1. The patient was positioned prone on a Jackson frame. Great care was used to place the head and neck in a neutral position, with the ears at the level of the shoulders. Arms were positioned in a manner that prevented traction on the brachial plexus. Elbows were bent to 90 degrees and the volar surface of the forearms was placed on foam pads after shoulders were abducted 90 degrees with 30 degrees of forward flexion. The patient underwent posterior instrumentation and fusion with hybrid fixation from T1 to L1. From incision to closure, the total operative time was four hours. Her estimated blood loss was 500 mls, and she received 53 ml of cell-saver blood autofusion prior to wound closure. Her mean arterial blood pressure (MAP) ranged from 95 mm Hg at incision to 75 mmHg at closure, with a high of 95 mmHg and a low of 59 mmHg. Neurologic status was monitored using SSEPs and transcranial MEPs without evidence of intraoperative abnormalities throughout the procedure. Prior to the procedure, her Hg was 11.2 and this was not rechecked immediately postoperatively. At the completion of the procedure, she was rolled supine on a hospital bed where she actively moved all major joints of upper and lower extremities on command prior to extubation. She was taken to the recovery room where she was noted to have full upper extremity and lower extremity function. Her arterial line was removed, and she was transferred to the floor after one hour.

On arrival to the floor, the patient did complain of slight numbness/tingling in her knees; however, her exam was normal and this was attributed to her prone positioning during surgery with pressure on the anterior surface of the knees.

At 11 pm (12 hours after the procedure), the patient began complaining of weakness in her upper and lower extremities as well as decreased sensation in all extremities. The patient was anxious and had periods of apnea, likely due to anxiety and narcotic usage. Her MAPs were between 62 and 65.

Examination at this time revealed the patient was anxious and tachypneic. She was unable to move her lower extremities bilaterally (0/5 in all major muscle groups) with decreased sensation in the L3-S1 distribution. Left upper extremity exam at this time was completely normal while the right upper extremity demonstrated decreased sensation in C5 and T1 along with difficulty moving fingers and wrist on the right side (3/5 wrist flexion, wrist extension, and grip strength). The patient was given 1.5L normal saline at this time to help raise her blood pressure. Her hemoglobin at this time was 10.1.

Two hours after the onset of symptoms, the patient regained full strength and sensation in bilateral lower extremities and in her left upper extremity. Her right upper extremity regained full sensation, however, she continued to have weakness with elbow, finger, and thumb extension, as well as grip strength (all 3/5).

**Figure 2:**
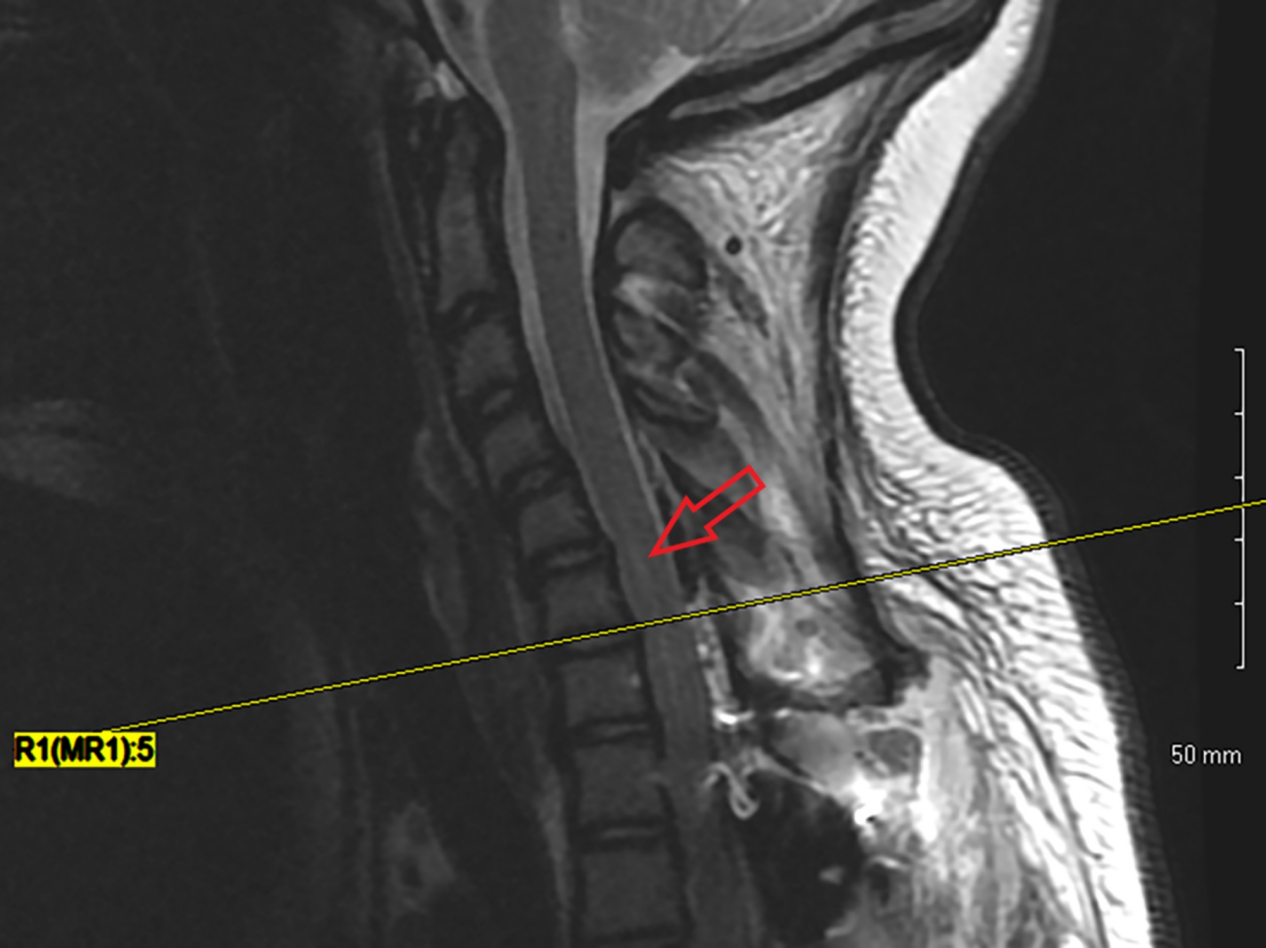
Postoperative sagittal MRI demonstrating acute myelomalacia changes of the spinal cord

**Figure 3:**
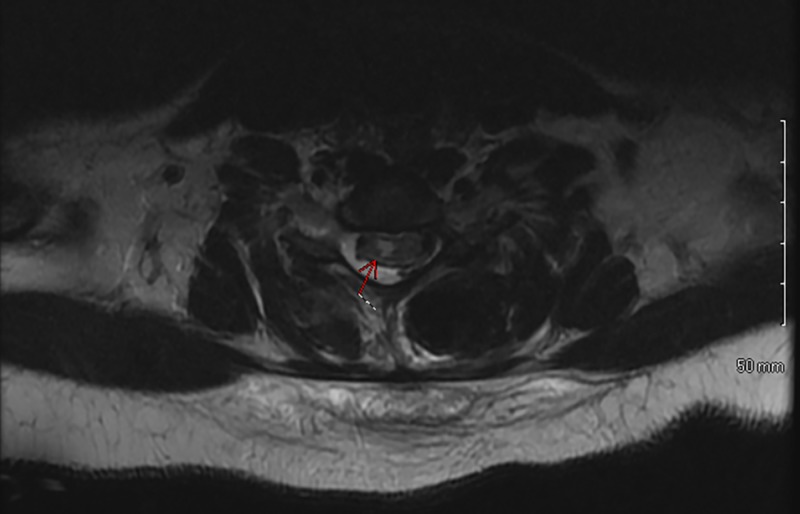
Postoperative axial MRI demonstrating acute myelomalacia changes of the spinal cord MRI: magnetic resonance imaging

An MRI was subsequently obtained, which demonstrated a 4.2 cm longitudinal right-sided hemicord infarction from C5-C7 (Figures 2-3).

The patient was immediately transferred to the pediatric intensive care unit (PICU) and started on methylprednisolone and a norepinephrine drip in order to maintain MAP >70.

Before discharge from the hospital, neurologic symptoms began to improve. She started having gradual improvement of right triceps function, however, she was still unable to fire finger or thumb extensors. She had full motor and sensory function in her left upper extremity and in her bilateral lower extremities.

**Figure 4:**
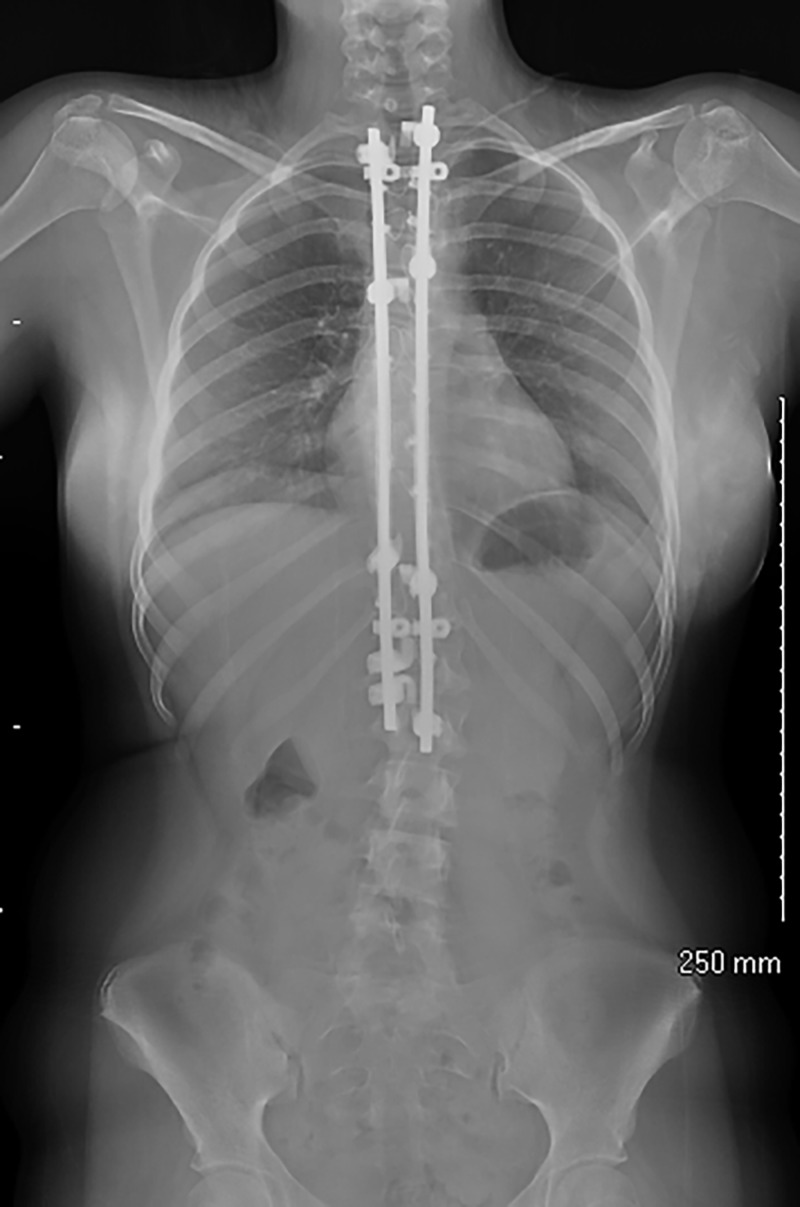
Postoperative AP radiograph demonstrating postoperative correction of scoliosis AP: anteroposterior

At one month from the surgery, the patient had regained full sensation throughout her right arm from the C4-T1 distributions. Her strength on her right side was: biceps 5/5, triceps 4+/5, wrist extensors, grip strength, and extensor pollicis longus 4/5, and extensor digitorum communis and extensor indices 2+/5. Occupational therapy was started to work on strengthening and motion for her right arm. At four months from her surgery, she demonstrated improved coronal alignment but normal sagittal balance (Figure 4).

Her sensation remained intact in the right arm. Motor function demonstrated full grip strength and triceps function, mild weakness of her extensor digitorum communis (EDC) and extensor indicis proprius (EIP), and the inability to fully extend her fingers in her right hand. Occupational therapy was continued.

## Discussion

Posterior spinal fusion is a safe and reliable method to treat AIS. Neurological complications occur at a rate of approximately 0.13% to 1% [1-3]. The majority of these can be attributed to a direct injury to the cord or traction injuries due to intraoperative stretch. These injuries are usually detected intraoperatively with changes in SSEP and MEPs. If the injury is persistent, it is confirmed in the immediate postoperative period based on a physical examination. Using advanced imaging to locate the level and degree of injury is usually difficult to evaluate due to the metal artifact within the instrumented portions of the thoracic and lumbar spine. MRI can help evaluate the status of the cord in non-instrumented areas.

This case is unusual because the patient had normal intraoperative spinal cord monitoring and in the immediate postoperative period, she was neurologically intact. These facts, in addition to the shorter surgical length, relatively low blood loss, and moderate surgical correction make it unlikely that the injury is due to an intraoperative event and likely occurred in the evening of the operative day.

The MRI findings demonstrated a zone of injury significantly above the level of the deformity and surgical correction and exclusively on the right side, which may have explained the initial symptoms and the reason why her left upper extremity neurological exam remained within normal limits. We hypothesize that this injury was caused by ischemia due to hypoventilation from narcotic use and/or hypoperfusion secondary to low MAP and hemoglobin. We suspect these factors can be compounded by altered vascular anatomy in the neck as a result of mechanical changes in the cord from surgical correction and possibly head positioning while she slept, as her cervical spine was not immobilized with a brace.

Similar findings of cervical ischemia after thoracolumbar posterior spinal fusion have been reported previously [6-7]. Some of those cases had an epidural catheter placed. In general, epidural use for pain control has fallen out of favor as injury in the cervical and thoracic spinal cord region may have occurred as a result of volume in the epidural space or changes in spinal cord physiology from the pharmacologic agents used in the epidural [8]. It has been hard to prove that spinal cord injury is a direct result of epidural use, yet our institution has abandoned its use despite a prior publication from our institution that expounds its benefits [9]. The patient in this report did not have an epidural placed.

In a recently reported series of patients with DPND, 90% occurred within the first 48 hours; the majority had complete or partial recovery while up to a third had no recovery [5]. It is important to note that, in this series, compression-related DPND had a greater likelihood of experiencing some neurological recovery as compared to ischemia-related DPND.

In summary, we report the case of a patient who had uneventful PSF for AIS and whose intraoperative spinal cord monitoring and immediate postoperative exam suggested no surgically related injury to her spinal cord. Yet, 12 hours later, she developed symptoms thought to be secondary to C5-7 myelomalacia. We suspect a combination of factors such as an ischemic injury as a result of hypoxia from hypoventilation, hypotension, and changes in the microvascular anatomy from changes in cord length from the spinal correction.

Our hypothesis is impossible to prove, yet the occurrence of delayed spinal cord injury at all levels is possible and demands vigilance for changes in the postoperative period. Surgeons, residents, nurses, and ancillary staff should be made aware of this rare complication and be ready to address it immediately. If changes in neurologic function are noted, steps to optimize conditions for the cord can be taken such as optimizing oxygenation, blood pressure, and hematocrit.

## Conclusions

This is a case report of a cervical spine injury following a posterior thoracic instrumented fusion. The aim is to make clinicians aware of this rarely reported complication following adolescent idiopathic scoliosis surgery. The risk persists despite not using an epidural and can occur with no changes in neuromonitoring during the procedure. The exact etiology is unknown but is likely due to hypotension at the cord level.
